# Divergent Longitudinal MRI Trajectories in First-Episode Schizophrenia: Evidence for Distinct Disease Subtypes?

**DOI:** 10.1192/j.eurpsy.2026.12130

**Published:** 2026-07-31

**Authors:** F. Spaniel, V. Čapek, A. Skoch, M. Kolenic

**Affiliations:** Psychaitry, National Institute of Mental Health, Klecany, Czech Republic

## Abstract

**Introduction:**

Schizophrenia is a severe psychiatric disorder characterized by complex alterations in brain structure and function, particularly during its early stages. Despite decades of research, the biological mechanisms driving these cerebral changes remain only partially understood. Emerging evidence suggests that neuroinflammatory processes and structural brain abnormalities may play convergent roles in disease progression. To elucidate these mechanisms, we leveraged data from one of the largest longitudinal investigations of first-episode schizophrenia.

**Objectives:**

This study aimed to characterize longitudinal trajectories of cortical architecture in first-episode schizophrenia and to determine whether neuroinflammatory mechanisms, particularly those mediated by innate immune responses, contribute to the observed structural changes.

**Methods:**

Data were derived from the **ESO study**, a large-scale longitudinal project involving **over 200 patients with first-episode schizophrenia** and **120 healthy control subjects**. Participants underwent repeated multimodal MRI assessments and clinical evaluations during the first years following illness onset. Structural MRI data were analyzed to identify patterns of cortical thickness and surface changes over time, and these were examined in relation to markers of neuroinflammation and immune system activation.

**Results:**

Analysis revealed **distinct trajectories in cortical architecture** among patients, indicating considerable heterogeneity in structural brain alterations. Despite these divergent patterns, a **convergent mechanism of neuroinflammation**, primarily driven by **innate immune responses**, was consistently associated with early-stage schizophrenia. These findings provide new insight into the interplay between immune dysregulation and neuroanatomical changes, supporting the view that schizophrenia may adhere to **fundamental principles of neurodegeneration**.

**Conclusions:**

Findings from this study **suggest** that schizophrenia is a biologically heterogeneous disorder, with diverse structural trajectories that may **converge on shared neuroinflammatory and neurodegenerative processes**.This work was supported by the Operational Programme Jan Amos Komenský (OP JAK), grant no. CZ.02.01.01/00/23_020/0008560

**Disclosure of Interest:**

None Declared

